# The impact of the COVID-19 pandemic on nosocomial infections: a retrospective analysis in a tertiary maternal and child healthcare hospital

**DOI:** 10.3389/fpubh.2023.1132323

**Published:** 2023-04-18

**Authors:** Huifang Huang, Kunhai Wu, Haiming Chen, Jing Wang, Lufei Chen, Zhirun Lai, Shuling Lin

**Affiliations:** ^1^Intensive Care Unit, Fujian Maternity and Child Health Hospital College of Clinical Medicine for Obstetrics & Gynecology and Pediatrics, Fujian Medical University, Fuzhou, Fujian, China; ^2^Blood Transfusion Department, Fujian Maternity and Child Health Hospital College of Clinical Medicine for Obstetrics & Gynecology and Pediatrics, Fujian Medical University, Fuzhou, Fujian, China; ^3^Department of Laboratory Medicine, The First Affiliated Hospital of Xiamen University, Xiamen Key Laboratory of Genetic Testing, Xiamen, China; ^4^Department of Infection Control, Fujian Maternity and Child Health Hospital College of Clinical Medicine for Obstetrics & Gynecology and Pediatrics, Fujian Medical University, Fuzhou, Fujian, China

**Keywords:** COVID-19 pandemic, nosocomial infection, infection rate, pathogen, epidemiology

## Abstract

**Objective:**

The constant changes in the control strategies of the Corona Virus Disease 2019 (COVID-19) pandemic have greatly affected the prevention and control of nosocomial infections (NIs). This study assessed the impact of these control strategies on the surveillance of NIs in a regional maternity hospital during the COVID-19 pandemic.

**Methods:**

This retrospective study compared the observation indicators of nosocomial infections and their changing trends in the hospital before and during the COVID-19 pandemic.

**Results:**

In total, 2,56,092 patients were admitted to the hospital during the study. During the COVID-19 pandemic, the main drug-resistant bacteria in hospitals were *Escherichia coli, Streptococcus agalactiae, Staphylococcus aureus, Klebsiella pneumoniae*, and Enterococcus *faecalis*. The detection rate of *S. agalactiae* increased annually, while that of *E. faecalis* remained the same. The detection rate of multidrug-resistant bacteria decreased during the pandemic (16.86 vs. 11.42%), especially that of CRKP (carbapenem-resistant *Klebsiella pneumoniae* 13.14 vs. 4.39, *P* < 0.001). The incidence of nosocomial infections in the pediatric surgery department decreased significantly (OR: 2.031, 95% CI: 1.405–2.934, *P* < 0.001). Regarding the source of infection, a significant reduction was observed in respiratory infections, followed by gastrointestinal infections. In the routine monitoring of the ICU, the incidence of central line-associated bloodstream infection (CLABSI) decreased significantly (9.4/1,000 catheter days vs. 2.2/1,000 catheter days, *P* < 0.001).

**Conclusion:**

The incidence of nosocomial infections was lower than that before the COVID-19 pandemic. The prevention and control measures for the COVID-19 pandemic have reduced the number of nosocomial infections, especially respiratory, gastrointestinal, and catheter-related infections.

## 1. Introduction

Nosocomial infections (NIs), also known as hospital-acquired infections, are infections occurring within 48 h of hospital admission, within 30 days after receiving healthcare, or up to 90 days after undergoing surgery (1). NIs can prolong hospitalization and increase antimicrobial resistance, medical expenses, and mortality rates. NIs affect ~2 million individuals in the United States annually, with 80,000 of them dying as a result, resulting in economic losses of more than $4.6 billion (2). In a tertiary hospital in China, the average hospital stay was longer, and hospitalization costs were higher in patients with multidrug resistance (MDR) (3).

Until now, many countries have established their national or regional nosocomial infection surveillance systems (4–7). A systematic review (8) showed that continuous surveillance had a positive impact on NI, and OR/RR ranged from 0.43 to 0.95. However, in maternity hospitals, patient composition, disease severity, and common pathogens varied from those in general hospitals (9, 10). It is important to understand the current situation of NIs in maternity hospitals to guide further prevention and control.

The global COVID-19 pandemic has further aggravated the severity of NIs. The rate of methicillin-resistant *Staphylococcus aureus* (MRSA) infections among hospitalized patients during the COVID-19 outbreak was substantially greater in SARS-CoV-2 positive patients than in virus-negative patients (11). There was a significant increase in the incidence of New Delhi metallo-β-lactamase-producing carbapenem-resistant Enterobacterales and carbapenem-resistant *Acinetobacter baumannii* at some hospital sites when compared with that before the COVID-19 pandemic (12). Hospitals at all levels have strengthened the prevention of COVID-19 transmission and control of NIs.

In the study hospital, strategies were developed to prevent hospital-associated transmission of COVID-19. The outpatient volume was controlled, and screening processes were strictly performed. Emergency surgeries were performed in a negative-pressure room to protect the surgeons. All medical staff, including the logistics staff, received extensive training on awareness and prevention of NIs, which comprised two major parts: theoretical training and skill training. A new computer system was used to record surveillance data and provide timely feedback. However, similar data on the influence of the COVID-19 pandemic on NIs in maternity hospitals have not yet been reported. This study assessed the impact of these control strategies on NI surveillance in a regional maternity hospital during the COVID-19 pandemic.

## 2. Material and methods

### 2.1. Study design and sample size

This retrospective study was conducted at a large tertiary maternity hospital in Fuzhou, China between 1 January 2018 and 31 December 2021. The hospital serves as both a tertiary maternity hospital in the Fuzhou region and a tertiary referral center for complicated maternal, pediatric, and neonatal cases. A total of 2,56,092 patients were admitted to the hospital during the study period.

Surveys conducted from 1 January 2018 to 31 December 2019 were defined as the pre-pandemic group, while those conducted from 1 January 2020 to 31 December 2021 were defined as the during-pandemic group.

### 2.2. Ethics approval

This study was approved by the Ethics Committee of Fujian Maternity and Child Health Hospital College of Clinical Medicine for Obstetrics and Gynecology and Pediatrics, Fujian Medical University (ethics approval number: 2022YJ070). Because this retrospective analysis used routinely collected data, the requirement for informed consent was waived.

### 2.3. Inclusion and exclusion criteria

The inclusion criteria were as follows:

- Patients admitted to all clinical wards who were present at the time of the survey were included.

The exclusion criteria were as follows:

- All patients who were infected at the time of admission or within 48 h after hospitalization were excluded.
- Patients with duplicate isolates, defined as the same bacteria isolated from the same patient and sample, were excluded.

### 2.4. Data collection

Identification of NIs was done by (1) nosocomial infection-warning systems triggered in the laboratory, (2) clinicians entering data into a hospital-based data system, and (3) confirmation by staff members of the hospital's infection control department. The diagnostic criteria for NIs were based on the diagnostic criteria for nosocomial infections developed by the Ministry of Health of the People's Republic of China (13), which is a modification of the definition provided by the US Center for Disease Control and Prevention (14).

The device-associated infection (DAI) incidence rate per 1,000 device days was calculated as the total number of DAIs divided by the total number of specific device days (days of indwelling urinary catheterization, central line use, or ventilator treatment) and multiplied by 1,000. DAIs included catheter-associated urinary tract infections (CAUTI), central line-associated bloodstream infections (CLABSI), and ventilator-associated pneumonia (VAP).

Clinical data, including information on pathogenic microorganisms, distribution in clinical wards, source of the infection, and DAIs, were extracted from the laboratory information system and the hospital-based nosocomial infection surveillance system.

### 2.5. Statistical analysis

All statistical analyses were performed using SPSS Statistics software (version 25.0; IBM Corp., Armonk, NY, USA). Descriptive statistics are reported as numbers (percentages) for categorical data. Chi-square or Fisher's exact tests were used to compare discrete variables. Fisher's exact test was used when **one** or more expected values were equal to or < 5. Binary logistic regression was used to analyze the relevant data. Statistical significance was set at a *p*-value of < 0.05. *A priori* sample size calculations were not performed because of the retrospective nature of the analysis, and all eligible cases were included.

## 3. Results

**Figure 1:**
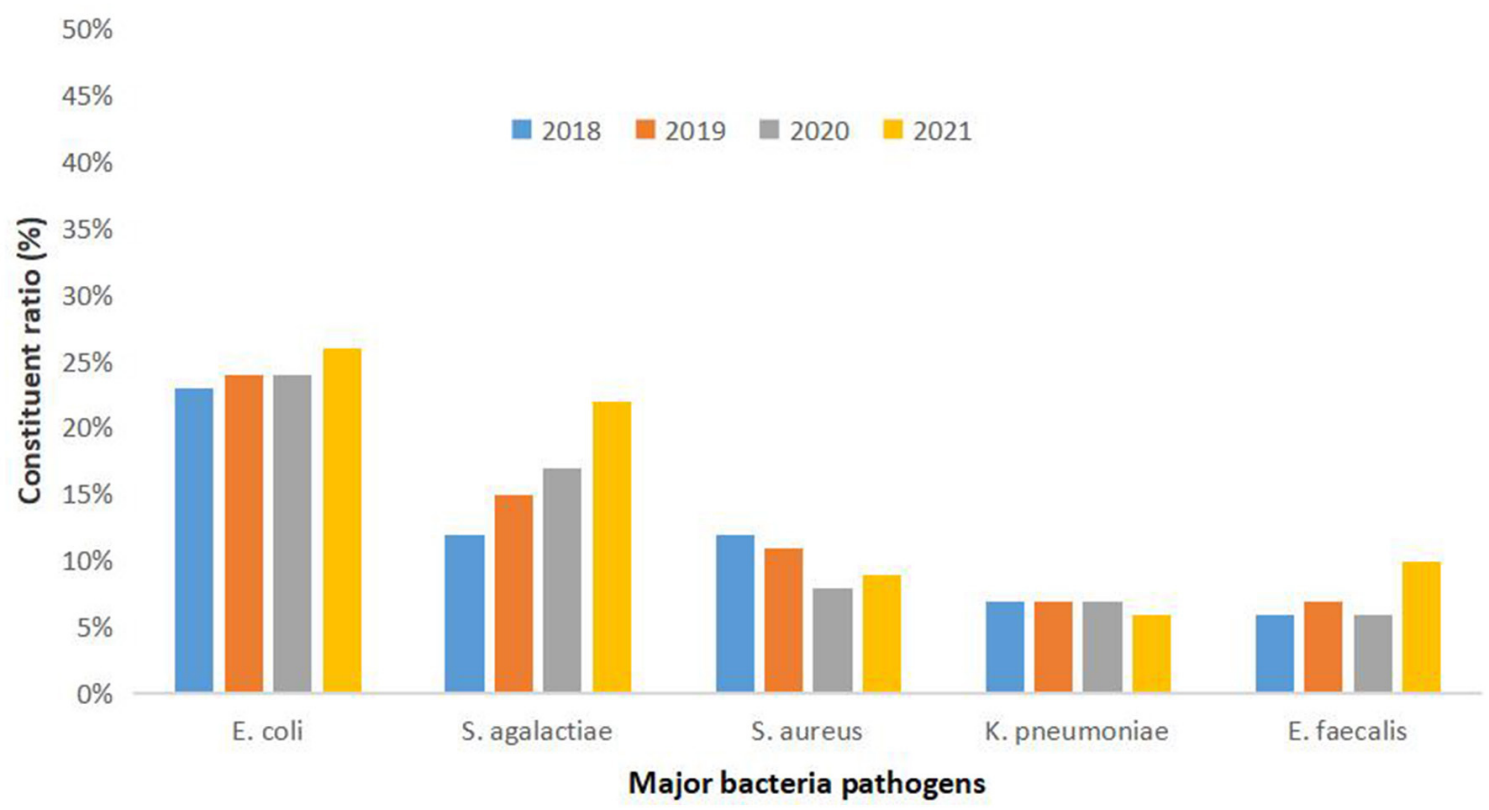
Distribution of the most prevalent drug-resistant bacteria.

Between January 2018 and December 2021, 2,56,092 patients were admitted to our hospital. In cultures taken from patients who were suspected of infection or before antibiotic administration, 8,043 samples tested positive for drug resistance (*n* = 4,187 in the pre-pandemic group and *n* = 3,856 in the during-pandemic group; Table 1). A detailed analysis of the pathogens showed that *Escherichia coli, Streptococcus agalactiae, Staphylococcus aureus, Klebsiella pneumoniae*, and *Enterococcus faecalis* were the most prevalent drug-resistant bacteria in NIs in the last 4 years, and the distribution was similar every year. The detection rate of *S. agalactiae* increased annually (from 12% in 2018 to 22% in 2022) (Figure 1).

**Table 1:** Observed infection rates (%) of drug-resistant pathogens.

**Drug-resistant pathogens**	**Pre-pandemic group, *n* = 4,187**	**During-pandemic group, *n* = 3,856**
*Escherichia coli*	23.36	24.66
*Streptococcus agalactiae*	13.83	19.14
*Staphylococcus aureus*	11.25	8.45
*Klebsiella pneumoniae*	7.17	6.25
*Enterococcus faecalis*	6.38	8.12

Multidrug-resistant bacteria such as methicillin-resistant *Staphylococcus aureus* (MRSA), carbapenem-resistant *Acinetobacter baumannii* (CRAB), carbapenem-resistant *Pseudomonas aeruginosa* (CRPA), carbapenem-resistant *Klebsiella pneumoniae* (CRKP), and carbapenem-resistant *Escherichia coli* (CRE-CO) were detected during the study period. The total detection rate of multidrug-resistant bacteria was lower during the COVID-19 pandemic than before (16.86%, pre-pandemic; 11.42%, during-pandemic). Among them, CRKP occurred at a significantly lower rate (*P* < 0.001) than that before the pandemic (Table 2).

**Table 2:** Detection rate of multi-drug resistant bacteria pathogens (%).

**Bacterial species**	**Pre-pandemic group**		**During-pandemic group**		***P***
	**Positive samples**	**Samples taken (%)**	**Positive samples**	**Samples taken (%)**	
MRSA	192	463 (41.47)	122	340 (35.88)	0.089
CRAB	27	70 (38.57)	51	95 (53.68)	0.055
CRPA	15	82 (18.29)	8	86 (9.30)	0.090
CRKP	41	312 (13.14)	10	228 (4.39)	< 0.001
CRE-CO	9	923 (0.98)	12	1,026 (1.17)	0.678
Total	284	1,684 (16.86)	203	1,778 (11.42)	< 0.001

MRSA, methicillin-resistant Staphylococcus aureus; CRAB, carbapenem-resistant Acinetobacter baumannii; CRPA, carbapenem-resistant Pseudomonas aeruginosa; CRKP, carbapenem-resistant Klebsiella pneumonia; CRE-CO, carbapenem-resistant Escherichia coli.

In this study, the incidence of nosocomial infections was lower than the national average. The decline in the prevalence of NIs was most pronounced in the pediatric surgery department. The prevalence was 1.17% (85/7,283) before the pandemic and 0.58% (43/7,437) during the pandemic in the pediatric surgery department. The prevalence of NIs in the other departments during the pandemic did not differ significantly from that before the pandemic (Table 3).

**Table 3:** The prevalence of NIs in different departments.

**Departments**	**Pre-pandemic group**		**During-pandemic group**		***P***	**OR**	**95%CI**
	**Infection cases**	**Cases investigated (%)**	**Infection cases**	**Cases investigated (%)**			
Total	515	1,33,439 (0.38)	434	1,22,653 (0.35)	0.182	1.091	0.960–1.240
Obstetrics	124	50,579 (0.25)	120	47,337 (0.25)	0.794	0.967	0.752–1.243
Gynecology	84	27,546 (0.30)	90	30,628 (0.29)	0.807	1.038	0.771–1.398
Pediatric surgery	85	7,283 (1.17)	43	7,437 (0.58)	< 0.001	2.031	1.405–2.934
Pediatric	50	17,557 (0.28)	40	9,970 (0.40)	0.106	0.709	0.467–1.075
Neonatology	30	15,464 (0.19)	18	13,690 (0.13)	0.192	1.476	0.823–2.650
ICUs	142	14,393 (1.00)	119	11,545 (1.03)	0.723	0.957	0.749–1.222
Others	0	617 (0)	4	2,046 (0.20)	1	–#	–#

ICU, intensive care unit, ICUs was comprised of Obstetrics and Gynecology ICU, Pediatric ICU and Neonatal ICU; OR, odds ratio; CI, confidence interval.

^#^Using fisher exact probability method, OR and 95%CI cannot be calculated.

**Figure 2:**
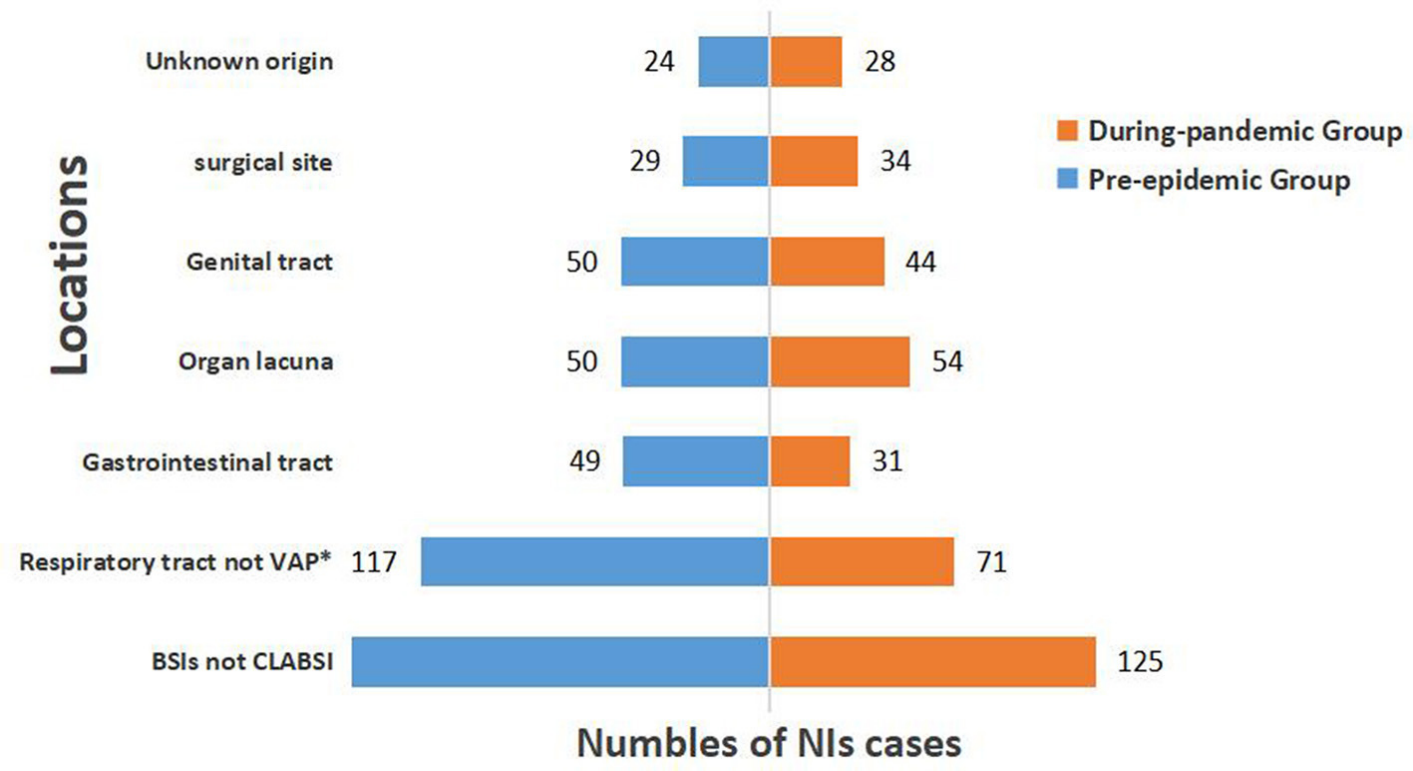
Distribution of locations of NIs before and during COVID-19 pandemic. VAP, ventilator-associated pneumonia; BSIs, bloodstream infections; CLABSI, central line associated-bloodstream infection; *Represents statistically significant difference (*P* < 0.05).

The most obvious decline in the pandemic group was observed in respiratory infections; however, the incidence of NIs at other sites during the pandemic did not change significantly. The tendency declined in gastrointestinal infections, genital tract infections, and bloodstream infections (BSIs) but not in CLABSI; however, there was no statistically significant difference between the two groups (Figure 2).

Device-associated infections (DAIs) are another specific type of NI, and these patients were mostly admitted to the ICU. The incidence rates of ventilator-associated pneumonia (VAP), central line-associated bloodstream infection (CLABSI), and catheter-associated urinary tract infection (CAUTI) were assessed at three ICUs in the study hospital. The incidence of CLABSI during the COVID-19 pandemic decreased significantly compared to that before the pandemic (9.4/1,000 catheterization days vs. 2.2/1,000 catheterization days, *P* < 0.001) (Table 4).

**Table 4:** The target monitoring of DAIs in ICUs.

**Device-associated infection**	**Pre-pandemic group**	**During-pandemic group**	***P***	**OR**	**95%CI**
VAP (1/1,000 Ventilator days)	11/892	19/815	0.090	0.523	0.247–1.106
CLABSI (1/1,000 Cath-days)	29/3,090	13/5,967	< 0.001	4.339	2.252–8.359
CAUTI (1/1,000 Cath-days)	13/18,628	15/18,975	0.742	0.883	0.420–1.856

DAIs, device-associated infection; VAP, ventilator-associated pneumonia; CLABSI, central line associated-bloodstream infection; CAUTI, catheter-associated urinary tract infection; Cath, catheter.

## 4. Discussion

Corona Virus Disease 2019 is characterized by rapid transmission, a wide range of infections, and great difficulty in prevention and control, seriously threatening human health and life (15). As the pandemic continues to change, work in hospitals has been greatly affected, especially hospital infection prevention and control work (16). During the post-pandemic period, knowledge of the composition of common pathogens and surveillance of NIs in hospitals will assist clinicians in making better decisions (17). However, previous studies have reported results mostly in tertiary general hospitals or a single department, such as the pediatric and ICU departments, with fewer data available for maternity hospitals (18–20). In this study, which was conducted in a tertiary maternity hospital, we found that between 2020 and 2021, the distribution of the main drug-resistant bacteria was similar to that before, and the detection rate of *S. agalactiae* increased annually. The detection rate of multidrug-resistant bacteria decreased during the pandemic, especially that of CRKP. In addition, during the pandemic, the incidence of NIs in the entire hospital was much lower than the national average, and the incidence in the pediatric surgery department decreased significantly. The incidences of VAP, CLABSI, and CAUTI were low, and the incidence of CLABSI decreased significantly.

Increased bacterial resistance is often associated with the overuse of antibiotics (21). A systematic analysis showed that there were an estimated 4.95 million deaths associated with bacterial antimicrobial resistance (AMR) in 2019 (22). During the study period, the top five drug-resistant bacteria were *Escherichia coli, Streptococcus agalactiae, Staphylococcus aureus, Klebsiella pneumoniae*, and *Enterococcus faecalis*. This is consistent with data from Ireland, where the common maternal pathogens were *E. coli* and Group B *Streptococcus* (10). However, unlike the top five bacteria detected in the national reporting data, the ones detected in this study were *Escherichia coli, Klebsiella pneumoniae, Staphylococcus aureus, Pseudomonas aeruginosa*, and *Acinetobacter baumannii* (7). This may be because no COVID-19 patients had been admitted to the study hospital, and the bacterial composition was mainly related to the environment, season, and composition of the patient population (women, children, and newborns). The prevalence of maternal colonization by *Streptococcus agalactiae* (Group B *Streptococci* [GBS]) varies according to the region studied and is approximately between 15 and 30% abroad (23), similar to our results. As a new consensus in China was released, targeted screening was added to the study hospital, and the detection rate of GBS increased annually.

During the COVID-19 pandemic, hospital administrators implemented some measures, such as better management of antibiotic use, better training for physicians, and better environmental hygiene. The detection rate of multidrug-resistant bacteria decreased from 18.86% before the pandemic to 11.42% during the pandemic; among them, methicillin-resistant *Staphylococcus aureus* (MRSA) was still the most detected bacterium, and the detection rate of CRKP decreased significantly. This declining trend differs from those observed in recent studies. A comprehensive review reported that, in recent years, CR-hvKP (Carbapenem-resistant Hypervirulent *Klebsiella pneumoniae*) has been increasingly reported in China, the United States, India, Russia, Egypt, Italy, and other countries, represented by the ST11, ST23, and ST258 types (24). Further analysis revealed that CRKP was mainly detected in children with severe disease in the study hospital. However, during the COVID-19 pandemic, pediatric hospitalizations decreased significantly, and antibiotic use was rigorously regulated, which would likely lead to a significant drop in detection rates. NIs caused by CRKP among children are becoming common (25), and it is necessary for pediatric wards to conduct CRKP screening on admission and standardize antibiotic use, which may decrease the incidence of CRKP infections.

With increasing awareness of nosocomial infection prevention, the prevalence rate of NIs in China decreased from 5.36% in 2001 to 1.98% in 2018 (26). During the study period, the incidence of NIs in hospitals was much lower than the national average. Since 2020, a series of recommendations have been made for the management of COVID-19 (27). Based on these guidelines, hospitals have developed hospital-specific strategies to guide clinical departments in their COVID-19 prevention efforts. However, few studies have investigated the relationship between interventions and nosocomial infections. In this study, we observed that NIs in the pediatric surgery department decreased from 1.17 to 0.58% after the response prevention to the COVID-19 pandemic. The incidence of NIs declined in other departments, such as neonatology and gynecology, but the difference was not significant.

When we further studied the locations of NIs, we found the most significant decrease in respiratory infections, followed by gastrointestinal infections, which were related to health habits during the COVID-19 pandemic. In COVID-19 prevention efforts, masks are widely used coupled with adequate social distancing, diligent hand hygiene, and other proven prevention measures (28). These measures may have reduced the spread of respiratory and gastrointestinal infections.

There was a decreasing trend in the monitoring of DAIs in the ICUs during the study period. In particular, the decrease in CLABSI was statistically significant. Similar to a previous study, Costello et al. (29) showed that the education of physicians and nurses and real-time feedback on CLABSI data led to a decrease in CLABSI rates. These results indicate that these measures can be continuously implemented to bEtter prevent and control NIs.

We acknowledge that there were some limitations in the study. First, the data used in this study were retrieved from the hospital infection surveillance system; therefore, there may have been selection bias. Second, without further analysis of the patient's symptoms, recovery, and treatment, the advice provided to different departments will be limited. Third, we only studied the data from 2018 to 2021, and the short year span may lead to limitations in this study.

The incidence of nosocomial infections was lower than that before the COVID-19 pandemic. Active surveillance of MDR, generation of data on etiological agents and their antimicrobial susceptibility patterns, implementation of standard infection control protocols, and re-education of hospital staff are needed to control the occurrence of nosocomial infections.

## Data availability statement

The raw data supporting the conclusions of this article will be made available by the authors, without undue reservation.

## Author contributions

HH, KW, HC, and JW designed the study. ZL and SL analyzed the clinical data. HH and KW wrote the manuscript. LC revised the manuscript and supervised the study. All authors have seen and approved the manuscript and its submission.
